# Synthesis of Copper Nanowires Using Monoethanolamine and the Application in Transparent Conductive Films

**DOI:** 10.3390/nano15090638

**Published:** 2025-04-22

**Authors:** Xiangyun Zha, Depeng Gong, Wanyu Chen, Lili Wu, Chaocan Zhang

**Affiliations:** School of Materials Science and Engineering, Wuhan University of Technology, Wuhan 430070, China; zxy0108@whut.edu.cn (X.Z.); gdp@whut.edu.cn (D.G.); chenwanyu@whut.edu.cn (W.C.); polym_wl@whut.edu.cn (L.W.)

**Keywords:** copper nanowires, monoethanolamine, nanomorphology, transparent conductive film

## Abstract

Copper nanowires (Cu NWs) are considered a promising alternative to indium tin oxide (ITO) and silver nanowires (Ag NWs) due to their excellent electrical conductivity, mechanical properties, abundant reserves, and low cost. They have been widely applied in various optoelectronic devices. In this study, Cu NWs were synthesized using copper chloride (CuCl_2_) as the precursor, monoethanolamine (MEA) as the complexing agent, and hydrated hydrazine (N_2_H_4_) as the reducing agent under strongly alkaline conditions at 60 °C. Notably, this is the first time that MEA has been employed as a complexing agent in this synthesis method for Cu NWs. Through a series of experiments, the optimal conditions for the CuCl_2_–MEA–N_2_H_4_ system in Cu NWs synthesis were determined. This study revealed that the presence of amines plays a crucial role in nanowire formation, as the co-ordination of MEA with copper in this system provides selectivity for the nanowire growth direction. MEA prevents the excessive conversion of Cu(I) complexes into Cu_2_O octahedral precipitates and exhibits an adsorption effect during Cu NWs formation. The different adsorption tendencies of MEA at the nanowire ends and lateral surfaces, depending on its concentration, influence the growth of the Cu NWs, as directly reflected by changes in their diameter and length. At an MEA concentration of 210 mM, the synthesized Cu NWs have an average diameter of approximately 101 nm and a length of about 28 μm. To fabricate transparent conductive films, the Cu NW network was transferred onto a polyethylene terephthalate (PET) substrate by applying a pressure of 20 MPa using a tablet press to ensure strong adhesion between the Cu NW-coated mixed cellulose ester (MCE) filter membrane and the PET substrate. Subsequently, the MCE membrane was dissolved by acetone and isopropanol immersion. The resulting Cu NW transparent conductive film exhibited a sheet resistance of 52 Ω sq^−1^ with an optical transmittance of 86.7%.

## 1. Introduction

Transparent conductive films are key components in optoelectronic devices, offering functions such as information interaction [1], energy conversion and storage [2], and healthcare applications [3]. Indium tin oxide (ITO), as a typical material for transparent conductive films, suffers from drawbacks such as high cost, brittleness, poor flexibility, toxicity, and low stability, which limit its sustainable application in flexible electronic devices [4,5,6]. As a result, alternative materials with lower costs and similar performance have attracted widespread attention. Against this backdrop, films based on carbon nanotubes (CNTs) [7], graphene [8], and metal nanowires [9] have been widely investigated. Although CNTs and graphene intrinsically possess excellent charge transport properties, their macroscopic films often exhibit lower electrical conductivity than metal nanowire networks due to contact resistance at junctions within the conductive network [10]. Moreover, high-quality graphene typically relies on cost-intensive, low-throughput techniques such as chemical vapor deposition (CVD), and the fabrication process is complex, which hampers large-area production. Similarly, CNTs face challenges related to poor dispersibility and inconsistent diameters during synthesis and purification, limiting their uniformity and controllability [8,11]. In contrast, metal nanowires can be synthesized via solution-based methods under mild conditions (low temperature and atmospheric pressure), offering advantages of process scalability, cost-effectiveness, and maturity. Furthermore, metal nanowire networks exhibit high porosity and aspect ratios, enabling them to maintain excellent optical transparency and mechanical flexibility while ensuring high electrical conductivity. These properties make them more suitable than CNTs or graphene films for fulfilling the demanding requirements of high-performance transparent conductive films in flexible electronic applications [5,9].

Therefore, considering factors such as stability and conductivity, metal nanowire networks, particularly silver nanowires (Ag NWs) and copper nanowires (Cu NWs), exhibit excellent intrinsic conductivity and can decouple conductivity from optical transmittance, making them ideal alternatives to ITO [5,12]. For example, Ag NWs films have been demonstrated to possess electrical conductivity and optical transmittance comparable to ITO [13]. Moreover, copper is much more abundant than indium or silver and is more cost-effective. As a result, Cu NWs transparent conductive films can serve as a low-cost alternative to Ag NWs or ITO for transparent electrodes [14].

Existing synthesis methods for metal nanowires include ultraviolet light-assisted reduction [15], polyol method [16], liquid-phase reduction [17,18], and hydrothermal synthesis [19], with the most commonly used methods being hydrothermal synthesis and liquid-phase reduction. Hydrothermal synthesis involves the use of oleylamine (or octadecylamine, hexadecylamine, etc.) as a surfactant, with tris(trimethylsiloxy)silane (or glucose) as a reducing agent, under pressure at approximately 165 °C for 10 to 48 h [20,21]. Early studies extensively utilized long-chain alkylamines (such as oleylamine) to synthesize noble metal nanomaterials like gold, silver, and platinum in order to control the morphology and size of the nanoparticles [22,23]. Later, researchers further optimized the amount of long-chain alkylamine and reaction conditions and explored their applications in conductive materials [24,25,26]. Cu NWs synthesized via this method typically have diameters in the tens of nanometers and lengths ranging from a few micrometers to tens of micrometers. The transparent conductive films made from these Cu NWs usually exhibit a sheet resistance of 50–100 Ω sq^−1^ and a transmittance of around 80–90% [13,27,28,29]. This method, using long-chain alkylamines as solvents, offers strong controllability and scalability and allows Cu NWs to be obtained through self-reduction, ensuring good dispersion in the film-forming solution [30,31]. However, this approach often requires the addition of platinum, nickel, or other metal catalysts as initiators [32,33], which introduces impurities into the system, affecting the purity of the products and resulting in a lower yield of Cu NWs. Additionally, because Cu NWs are easily dispersed in organic solvents, their surfaces tend to adsorb organic molecules, which can adversely affect their conductivity in electronic devices.

The liquid-phase reduction method involves the use of ethylenediamine (EDA) as a complexing agent and hydrazine hydrate (N_2_H_4_) as a reducing agent in a strongly alkaline solution, reacting at temperatures below 80 °C for 1 h [34]. Cu NWs synthesized via this method typically have diameters in the range of 100–200 nm and lengths of several micrometers. The resulting transparent conductive films generally exhibit a sheet resistance of 30–100 Ω sq^−1^ and a transmittance of 85–95% [4,35,36]. Due to the strong complexing ability of EDA with Cu(II) compared to long-chain alkylamines like oleylamine and because this method allows for the rapid synthesis of Cu NWs at lower temperatures and under ambient pressure, it has been widely applied in mechanism studies. The existing literature has thoroughly investigated the synthesis mechanism of Cu NWs using this method, as well as the effects of the various substances and experimental parameters on the size and morphology of the Cu NWs. Wiley’s team [37] constructed a visualization system to observe the growth of Cu NWs and confirmed that Cu NWs grow through a diffusion-controlled reduction of copper(I) hydroxide, Cu(OH)_2_^−^. It was also demonstrated that EDA promotes the growth of Cu NWs by selectively inhibiting the formation of cuprous oxide (Cu_2_O) on the Cu(111) surface and enhancing the reduction rate of Cu(OH)_2_^−^ on the Cu(111) surface relative to Cu(100) [38]. Zhang et al. [39] also confirmed that the morphology of Cu NWs can be co-controlled by adjusting the stirring rate and varying the amounts of EDA and N_2_H_4_. However, as a strong ligand, EDA’s strong co-ordination ability can cause even slight differences to significantly affect the reduction process of Cu(II), which can hinder the uniformity of the size and the regularity of the morphology of Cu NWs [39,40,41]. Moreover, the high volatility and toxicity of EDA pose health risks to researchers and are not aligned with the growing trend of green and environmentally friendly practices. Additionally, copper sources commonly used in this method, such as copper nitrate, are chosen for their high solubility, which facilitates the rapid formation of a homogeneous copper ion solution, promoting reaction uniformity and controllability. Copper(II) in copper nitrate is easily reduced to Cu(0) by reducing agents such as N_2_H_4_ or ascorbic acid, allowing for faster reaction rates, making it suitable for the efficient synthesis of Cu NWs. However, the reduction reaction of copper nitrate is highly sensitive to pH, temperature, and reducing agent concentration, which complicates the control of Cu NW morphology. Furthermore, copper nitrate may release nitrogen oxides (NO_x_) during the reaction process, which negatively impacts its environmental friendliness.

Based on the aforementioned liquid-phase reduction method, this study replaces the complexing agent in the system from ethylenediamine (EDA) to monoethanolamine (MEA) and the copper source from copper nitrate to copper chloride. Compared to EDA, the complexing ability of MEA is relatively weaker, which means it does not excessively bind copper ions. However, under alkaline conditions, MEA can form relatively stable complexes with Cu(II) [42]. As a result, the copper ions involved in the reaction to form copper nanowires undergo a more moderate reaction rate, which helps control the concentration and reaction rate of copper ions in the system [43]. Research by Ellsworth et al. [44] on chemical plating indicates that using MEA can promote copper deposition on specific surfaces and control the growth direction and morphology of copper nanostructures. This suggests that MEA facilitates the directional growth of Cu NWs, promoting the formation of well-crystallized and structurally regular Cu NWs. (Note: ethanolamine (EOA) mentioned in reference [44] corresponds to the MEA used in this study.) Moreover, MEA is less expensive than EDA, has lower toxicity, lower volatility, and poses fewer health risks to researchers. It also generates less environmental pollution during disposal, aligning with the green and environmentally friendly development trend. In contrast to copper nitrate, copper chloride has a lower reduction potential for copper ions, making it easier to reduce to Cu(0) by reducing agents such as hydrazine hydrate or ascorbic acid, resulting in faster reaction rates. Additionally, copper chloride is more cost-effective, which facilitates large-scale synthesis of copper nanowires.

In this study, multiple sets of experiments were conducted by varying reaction temperature, sodium hydroxide concentration, MEA complexing agent concentration, and N_2_H_4_ concentration to determine the optimal experimental conditions and investigate the effects of these factors on the morphology and size of Cu NWs. The synthesis process, morphological structure, and the mechanism of action of the MEA complexing agent in the preparation of Cu NWs under the optimal conditions were also explored in detail. Furthermore, an improved vacuum filtration method was employed to prepare transparent conductive Cu NWs films.

## 2. Experimental Methods

### 2.1. Materials

Copper(II) chloride dihydrate (CuCl_2_·2H_2_O, 99%), sodium hydroxide (NaOH, 98%), monoethanolamine (MEA, 99%), and glacial acetic acid (C_2_H_4_O_2_, 99.8%) were purchased from Shanghai Macklin Biochemical Technology Co., Ltd. (Shanghai, China). Hydrazine hydrate (N_2_H_4_·xH_2_O, 80%), anhydrous ethanol (CH_3_CH_2_OH, 99.7%), polyvinylpyrrolidone (PVP, MW = 24 K), acetone (C_3_H_6_O, 99.5%), and isopropanol (C_3_H_8_O, 99.7%) were purchased from Aladdin Biochemical Technology Co., Ltd. (Shanghai, China). Polyethylene glycol 200 (PEG200) was purchased from Sinopharm Chemical Reagent Co., Ltd. (Shanghai, China). Mixed cellulose ester (MCE) membrane was purchased from Tianjin Jinteng Experimental Equipment Co., Ltd. (Tianjin, China). High-quality polyethylene terephthalate (PET) substrates were purchased from Hefei Microcrystal Material Technology Co., Ltd. (Hefei, China). Unless otherwise specified, all chemicals were used as received without further purification.

### 2.2. Preparation of Copper Nanowires

Based on previously reported synthesis methods [34,45], this study made slight modifications. A flask containing a NaOH aqueous solution (100 mL, 15 M) and CuCl_2_·2H_2_O (0.1 M, 5 mL) was preheated in a 60 °C oil bath for 30 min. Under vigorous stirring, a certain amount of MEA and hydrazine hydrate (10 M, 0.125 mL) solutions was added sequentially. After intense stirring for 2 min, stirring was stopped, and the reaction was continued under maintained temperature for 2 h. The solution began to precipitate a pink flocculent material within approximately 30 min, which rapidly rose to the surface of the liquid. The flocculent material formed a regular disc-shaped structure on the surface, gradually turning deep red. The copper nanowire discs were transferred to a centrifuge tube and washed by centrifugation twice with a 3 wt% N_2_H_4_ solution and once with anhydrous ethanol. The washed copper nanowires were then stored in a 3 wt% N_2_H_4_ solution for later use.

The PEG coating experiment was carried out by slowly injecting 10 mL of PEG along the inner wall of the flask using a pipette 10 min after adding the complexing agent and reducing agent, when the solution had completely clarified. The remaining experimental steps were carried out under normal experimental conditions. The washing solution for the product consisted of a 10 wt% PEG200 in N_2_H_4_ (3 wt%) solution, a pure N_2_H_4_ (3 wt%) solution, and anhydrous ethanol. The washed samples were stored in a N_2_H_4_ (3 wt%) solution containing 0 wt% PEG200 for future use.

### 2.3. Preparation of Transparent Conductive Films

The prepared and washed copper nanowires were dried in a 60 °C oven. A total of 5 mg of Cu NWs and 10 mg of PVP were dissolved in 100 mL of anhydrous ethanol and sonicated for 30–60 min to obtain a Cu NWs dispersion. The purchased PET substrates were cut into 2.5 × 2.5 cm^2^ pieces, soaked in piranha solution for 4 h, and then centrifugally washed 4 times with deionized water. After washing, the substrates were soaked in deionized water for later use.

**Figure 1:**
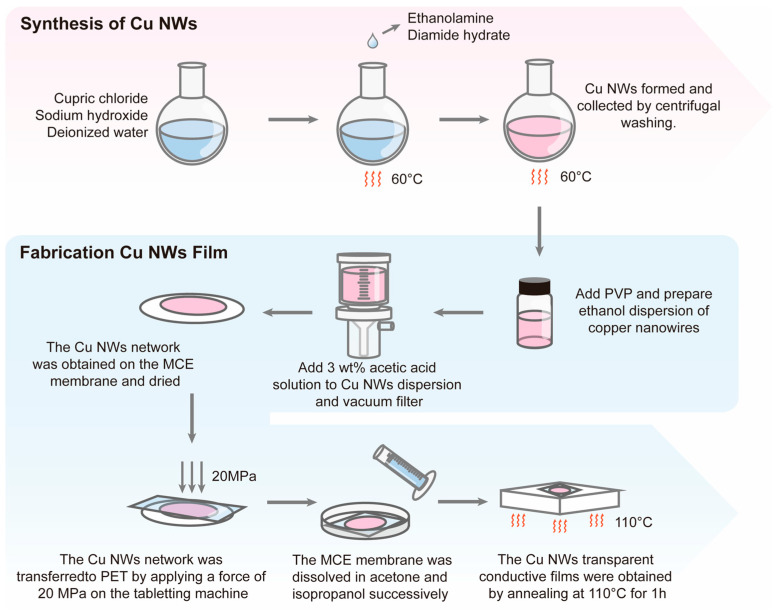
Schematic diagram of the synthesis of copper nanowires (Cu NWs) and the preparation of transparent conductive films.

A certain amount of the dispersion was added to 50 mL of acetic acid solution (3 wt%) and shaken to mix uniformly. Using a vacuum filtration method, a layer of Cu NWs network was formed on the MCE filter membrane (Figure 1). After allowing the membrane to rest for 20 min and air dry naturally, it was placed on the dried PET substrate. Under a pressure of 20 MPa applied by a press, the MCE filter membrane with the Cu NWs network was tightly bonded to the PET substrate. The membrane was then sequentially immersed in acetone and isopropanol, each for 30 min. After the soaking process, the membrane was removed and placed on a 110 °C heated platform for annealing for 1 h.

### 2.4. Material Characterization

The surface morphology and structure of the prepared products were observed using a Czech TESCAN MIRA LMS scanning electron microscope (TESCAN, Brno, Czech Republic) and an American FEI Tecnai G2 F20 field emission transmission electron microscope (FEI, Hillsboro, OR, USA). The crystalline phase of the products was characterized using a German Bruker D8 Advance X-ray diffractometer (Bruker, Leipzig, Germany) from Saarbrucken Technology GmbH (Saarbrücken, Germany). The chemical composition of the products was analyzed using an American Thermo Fisher Scientific ESCALAB 250Xi X-ray photoelectron spectrometer (Thermo Fisher Scientific, Waltham, MA, USA). The PerkinElmer Lambda 750-S UV-Vis spectrophotometer (200–1000 nm, Waltham, MA, USA) was employed to analyze the material composition and the optical transmittance of the transparent conductive films. The types and structures of the products and related materials were analyzed using a U.S. Horiba LabRAM Odyssey laser confocal micro-Raman spectrometer (He-Ne 633 nm, Irvine, CA, USA). The chemical composition of the products before and after coating was analyzed using an American Thermo Nicolet Nicolet 6700 Fourier-transform infrared (FTIR) spectrometer (400–4000 cm^−1^, Thermo Fisher Scientific, Waltham, MA, USA). The thermogravimetric curves (in air atmosphere) of the products before and after coating were measured using a German Netzsch STA 449 F3 integrated thermal analyzer (−150–2400 °C, Selb, Germany). The sheet resistance of the transparent conductive films was measured using a four-point probe tester (Model 0963307) manufactured by Ningbo Ruikeweiye Instruments Co., Ltd., Yuyao, China.

## 3. Results and Discussion

### 3.1. Synthesis of Cu NWs and Their Morphology and Structure

**Figure 2:**
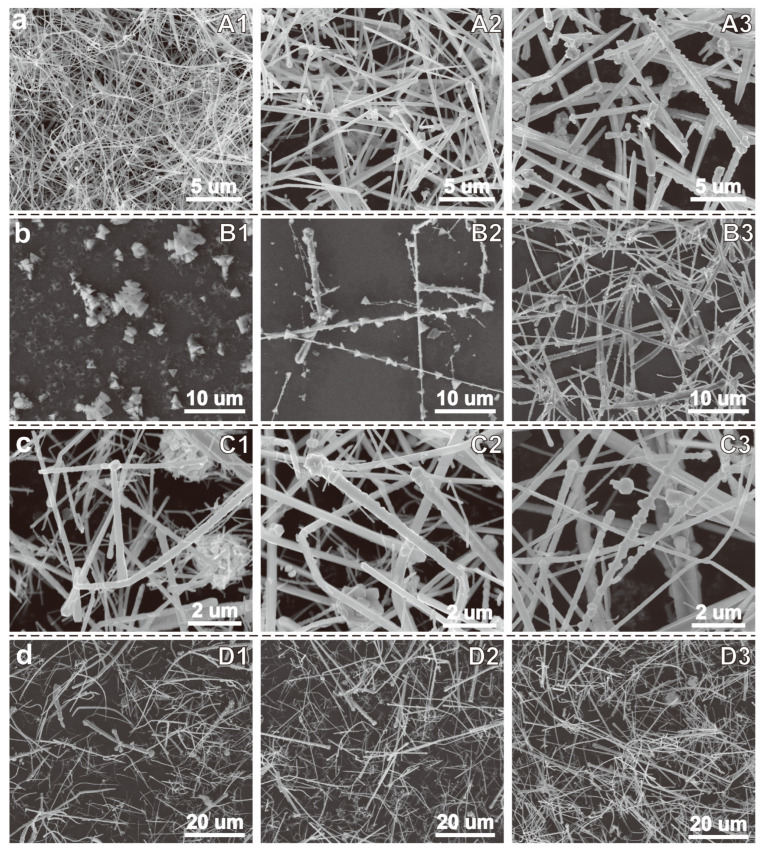
(**a**) SEM images of products synthesized at different temperatures (from left to right (A1–A3): 60 °C, 70 °C, and 80 °C); (**b**) SEM images of products synthesized with different NaOH concentrations (from left to right (B1–B3): 7.5 M, 10 M, and 12.5 M); (**c**) SEM images of products synthesized with different MEA concentrations (from left to right (C1–C3): 90 mM, 150 mM, and 300 mM); (**d**) SEM images of products synthesized with different N_2_H_4_ concentrations (from left to right (D1–D3): 7.5 mM, 10 mM, and 15 mM).

In this study, the copper source and complexing agent were replaced with more environmentally friendly and cost-effective copper chloride (CuCl_2_) and monoethanolamine (MEA), which likely led to significant changes in the reaction conditions. Therefore, we first screened four key parameters—reaction temperature, NaOH concentration, MEA concentration, and hydrazine hydrate (N_2_H_4_) concentration—to determine the optimal conditions for synthesizing Cu NWs. The experimental parameters and corresponding products are listed in Table 1, and the SEM images of the respective products are shown in Figure 2.

First, Cu NWs synthesis experiments were conducted at different temperatures. As shown in Figure 2a, at 60 °C, the obtained product consists of smooth-surfaced, uniform-sized Cu NWs (Sample A1). However, when the temperature was increased to 70 °C (Sample A2) and 80 °C (Sample A3), the nanowires exhibited a gradual increase in diameter and a decrease in length, with their surfaces becoming rougher. Therefore, the optimal reaction temperature was determined to be 60 °C. Subsequently, experiments were performed by varying the NaOH concentration (Figure 2b). When the NaOH concentration was 7.5 M, the reaction system remained transparent after 2 h. Even after extending the reaction time to 4 h, the solution only displayed a faint orange–yellow color. SEM images revealed that no nanowires were present in the product; instead, it consisted of agglomerated tetrahedral particles (Sample B1). XRD analysis confirmed that these particles were Cu_2_O tetrahedra (Figure S1). When the NaOH concentration was increased to 10 M, copper flakes were observed at the top of the reaction system. However, SEM images (Sample B2) indicated that the product was a mixture of nanowires and Cu_2_O tetrahedra. At a NaOH concentration of 12.5 M (Sample B3), the product primarily consisted of Cu NWs. However, compared to the Cu NWs obtained at 15 M NaOH (Sample A1), the nanowires synthesized at 12.5 M exhibited a larger and less uniform diameter distribution with shorter lengths. Therefore, the optimal NaOH concentration for the reaction was determined to be 15 M.

Figure 2c presents the SEM images of the products synthesized at different MEA concentrations. When the MEA concentration was 90 mM (Sample C1), the product consisted of aggregated nanoparticles and short, thick nanorods. As the MEA concentration increased to approximately 150 mM (Sample C2), the diameter of the nanoparticle aggregates decreased and the length of the nanorods increased. However, the nanorods were no longer short rods but rather elongated, tapered structures. When the MEA concentration reached 210 mM (Sample A1), the copper nanoparticles disappeared and the tapered rods further elongated into uniform copper nanowires with consistent thickness. At an MEA concentration of 300 mM (Sample C3), both the diameter and length of the Cu NWs slightly increased, but some particle accumulation was observed around the nanowires. Therefore, the optimal MEA concentration for the reaction was determined to be 210 mM.

Finally, we investigated the effect of N_2_H_4_ concentration on the reaction (Figure 2d). When the N_2_H_4_ concentration was 7.5 mM (Sample D1), the product mainly consisted of nanorods with irregular shapes and uneven diameters. At a N_2_H_4_ concentration of 10 mM (Sample D2), the nanorods exhibited reduced diameters and increased lengths, resulting in a mixture of nanorods and nanowires. When the N_2_H_4_ concentration reached 12.5 mM (Sample A1), the product displayed uniform morphology and well-defined dimensions. However, at a N_2_H_4_ concentration of 15 mM (Sample D3), the product showed increased diameters and reduced lengths, forming a mixture of nanorods and nanowires. Therefore, the optimal N_2_H_4_ concentration was determined to be 12.5 mM. The diameter and length distribution profiles of the samples from the above experiments are shown in Figure S2.

Based on the above experiments, we established the optimal conditions for the CuCl_2_–MEA–N_2_H_4_ system to synthesize Cu NWs, as represented by Sample A1. The best reaction parameters were determined to be 60 °C, NaOH concentration of 15 M, MEA concentration of 210 mM, and N_2_H_4_ concentration of 12.5 mM. In subsequent studies, we refer to these optimized conditions as A1.

**Figure 3:**
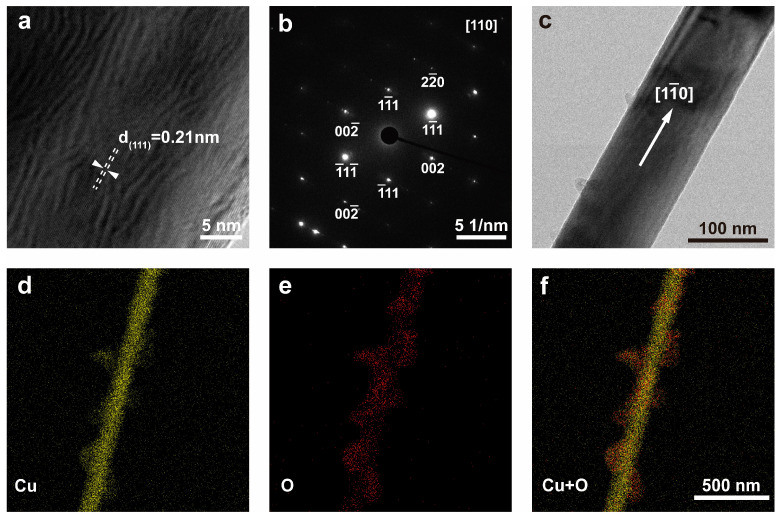
(**a**) HRTEM image of Cu NWs; (**b**) SAED pattern of Cu NWs; (**c**) overall HRTEM image of Cu NWs; (**d**–**f**) EDS mapping images of Cu NWs.

To gain a deeper understanding of the morphology and structure of Cu NWs, transmission electron microscopy (TEM) was employed to characterize the Cu NWs synthesized under the A1 conditions (Figure 3). The Cu NWs exhibited a smooth surface with uniform diameters, an average diameter of 101.34 nm, and an average length of approximately 27.86 μm (Figure S2a). The X-ray diffraction (XRD) pattern of the product confirmed that the nanowires possessed a face-centered cubic (FCC) copper structure (JCPDS 04-0836) (Figure S3). Figure 3a presents a high-resolution TEM image of the Cu NWs, where the calculated lattice spacing was 0.21 nm, corresponding to the (111) crystal plane of Cu. Figure 3b shows the selected area electron diffraction (SAED) pattern of the nanowire in Figure 3a, indicating that the Cu NWs grew along the (110) crystallographic direction (Figure 3c). We examined several other Cu nanowires and obtained the same SAED pattern, which differs from the fivefold-twinned Ag NWs and Cu NWs. Unlike those nanowires, our Cu NWs did not exhibit reflections from multiple zone axes, indicating that the synthesized Cu NWs are single-crystalline [16,21]. However, we cannot entirely rule out the possibility of stacking faults existing on the (111) planes [46].

Figure 3d–f present the EDS mapping images of the Cu NWs, confirming that the main structural component of the nanowire is Cu, indicating that the synthesized structure is indeed Cu NWs. However, it is noteworthy that the sheet-like structures surrounding the Cu NWs appeared during TEM imaging, as shown in Figure S4a. Combining the Cu and O elemental mapping images with the SAED pattern (Figure S4b), we identified these sheet-like structures as copper oxides. This phenomenon arises from the interaction between high-energy electrons and copper, which alters the electronic structure of Cu atoms, making them more susceptible to oxidation and forming copper oxides [47]. Additionally, the high-energy electron beam used in TEM imaging may modify the surface structure of Cu, increasing its surface area and potentially inducing nanoscale surface roughness. This increase in surface roughness enhances the contact area between Cu and oxygen, thereby accelerating the oxidation process.

### 3.2. Study on the Synthesis Process of Cu NWs

**Figure 4:**
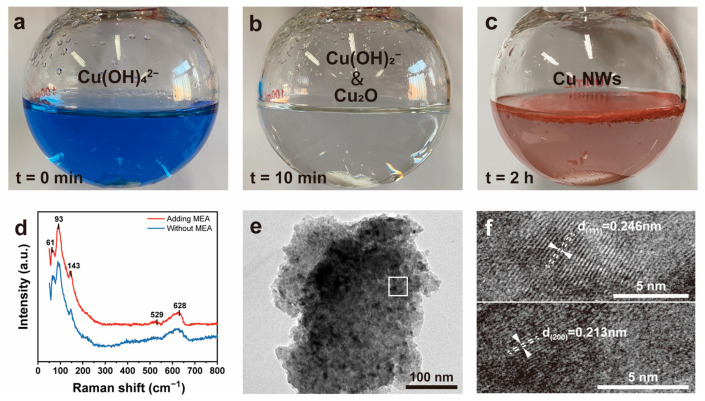
(**a**) Complex solution formed by CuCl_2_, NaOH, and MEA; (**b**) transparent solution formed after the addition of N_2_H_4_; (**c**) Cu NWs disk formed at the end of the reaction; (**d**) Raman spectra of the system with and without MEA at t = 10 min; (**e**,**f**) HRTEM images of Cu_2_O particles in the MEA-containing system at t = 10 min.

To investigate the transformation process of Cu(II) to Cu(0) in the CuCl_2_–MEA–N_2_H_4_ system, Raman spectroscopy and TEM analysis were conducted on the intermediate reaction stages. Figure 4a–c illustrate the color changes of the reaction over time. Upon adding CuCl_2_ solution and MEA to the NaOH solution, a deep blue complex formed (Figure 4a). After introducing the reducing agent N_2_H_4_, the solution transitioned through a milky-white phase before becoming clear and transparent (Figure 4b). This milky-white substance is almost certainly a Cu(I) complex, as Cu(I) remains stable at pH values greater than 10 within a temperature range of 25–300 °C [48]. To verify whether this reaction follows a mechanism similar to that of EDA complexation and whether trace amounts of Cu_2_O are present [49], further analyses were performed. After extracting the transparent solution from the reaction at the 5 min mark, we added a CH_3_COOH-CH_3_COONa buffer solution to quench the reaction and simultaneously lower the solution’s pH. The Raman scattering spectrum of the diluted solution was then acquired (Figure 4d). The most intense peak appeared near 93 cm^−1^, with two shoulder peaks at approximately 61 cm^−1^ and 143 cm^−1^. Additionally, two weak peaks were observed at 529 cm^−1^ and 628 cm^−1^ in the higher-frequency region. The Raman peaks at 62 cm^−1^ and 96 cm^−1^ correspond to the $\Gamma_{15}^{-}$ and $\Gamma_{25}^{-}$ vibrational modes, respectively [50]. The vibrations at 143 cm^−1^ and 628 cm^−1^ belong to infrared-active vibrational modes and can be attributed to $\Gamma_{15}$ modes induced by oxygen defects [51]. The peak at 529 cm^−1^ is close to the two-phonon mode reported in the literature [51]. However, the Raman peak at 219 cm^−1^ and the intrinsic peak at 595 cm^−1^ reported in the literature [51,52] were not observed. Additionally, all Raman peaks in Figure 4d exhibited a redshift of approximately 1–6 cm^−1^. To exclude the influence of MEA on the red shift of Cu_2_O Raman peaks, we performed Raman measurements on Cu_2_O octahedra synthesized under identical conditions without the addition of MEA. The results showed that the peak positions of the two spectra were identical, confirming that the red shift is unrelated to MEA. We attribute the red shift of the Cu_2_O Raman peaks in the system to changes in the solution pH, which affect the preferential orientation of Cu_2_O crystals. This leads to variations in the crystal structure and chemical bond strength of Cu_2_O, ultimately resulting in the observed red shift [53]. Furthermore, the high concentration of OH^−^ in the alkaline solution can cause the deprotonation of -NH_2_ groups adsorbed on the Cu_2_O surface, further contributing to the redshift of the Raman peaks [54].

Additionally, TEM analysis was performed on the diluted solution. Figure 4e shows Cu_2_O particle aggregates encapsulated by sodium acetate crystals, where the dark regions represent Cu_2_O particles. A high-resolution TEM (HRTEM) image corresponding to the boxed area in Figure 4e is shown in Figure 4f. The lattice fringes with an interplanar spacing of 0.246 nm correspond to the (111) plane of Cu_2_O, while those with an interplanar spacing of 0.213 nm correspond to the (200) plane of Cu_2_O. Based on Raman spectroscopy and TEM analysis, it can be confirmed that Cu_2_O particles, similar to those reported in the literature [55], appear in the MEA-complexed system after 10 min of reaction. This indicates that the reaction system using MEA as the complexing agent shares similarities with the system using EDA as the complexing agent in the synthesis of Cu NWs. In both cases, the reaction proceeds through an intermediate Cu(I) stage before being fully reduced to Cu(0) [49].

### 3.3. Investigation of the Mechanism of MEA as a Complexing Agent

**Figure 5:**
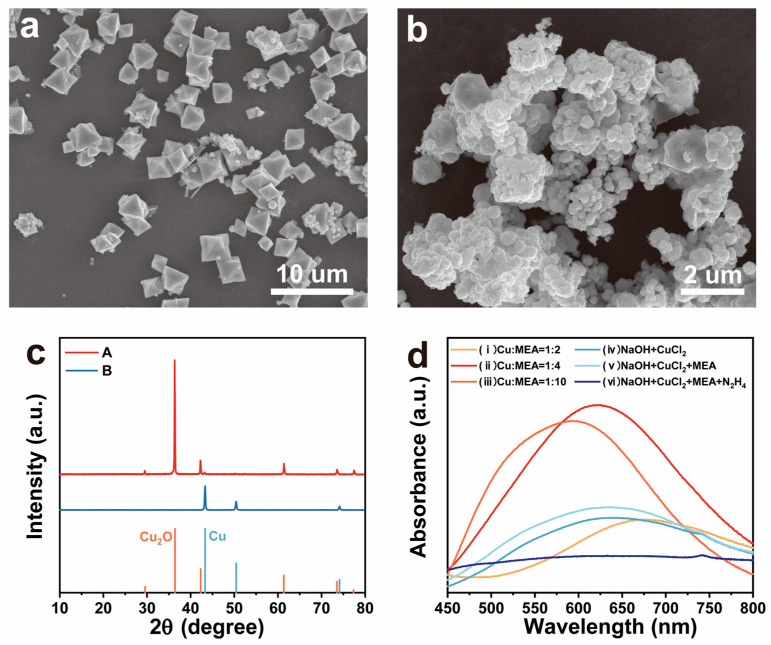
(**a**) SEM image of Cu_2_O particles formed in the absence of MEA; (**b**) reduction of Cu_2_O octahedra into irregular Cu particles in the absence of MEA; (**c**) XRD patterns corresponding to (**a**,**b**) with reference diffraction patterns at the bottom for Cu (JCPDS 04-0836, blue line) and Cu_2_O (JCPDS 05-0667, orange line); (**d**) UV-vis absorption spectra of Cu-MEA complex solutions with different Cu-to-MEA ratios and at different reaction stages.

MEA exhibits both amine and alcohol properties and, in the presence of Cu(II), it can co-ordinate with copper through amino, hydroxyl, and deprotonated hydroxyl groups, forming a copper–ethanolamine complex [56,57]. Therefore, to confirm the presence of the copper–ethanolamine complex during the reaction, understand the role of MEA in the Cu NWs growth process, and elucidate the growth mechanism of Cu NWs in this system, we varied the concentration of MEA and introduced a protective agent, followed by corresponding characterization of the products. As shown in Figure 5a, in the absence of MEA, Cu_2_O octahedra formed within approximately 5 min and the solution turned bright red. Further reduction of the Cu_2_O octahedra resulted in the formation of irregularly aggregated copper particles after about 2 h (Figure 5b). Figure 5c presents the XRD patterns of the Cu_2_O and Cu particles.

To confirm whether MEA participates in complexation after being introduced into the reaction system, UV-vis absorption measurements were conducted on three diluted mixed solutions with Cu-to-MEA molar ratios of 1:2, 1:4, and 1:10, as well as on solutions at three different stages before and after the reaction. The results are shown in Figure 5d. The absorption peaks of solutions (i)–(iii) exhibit a blue shift as the Cu-to-MEA ratio increases. Both solution (iv) and solution (v) show absorption peaks at 630 nm, which closely resemble the absorption peak of solution (ii). This indicates that, after the addition of MEA, the solution contains both the Cu(OH)_4_^2−^ complex and the CuCl_2_–MEA complex. The absorption peak at 630 nm in solution (iv) corresponds to the Cu(OH)_4_^2−^ complex formed by the reaction of CuCl_2_ with NaOH, while, in solution (v), after the addition of MEA, the peak at 630 nm corresponds to both the Cu(MEA)_4_(OH)_2_ complex and the Cu(OH)_4_^2−^ complex [43,58]. Upon the addition of N_2_H_4_, the solution becomes transparent and the absorption peak at 630 nm disappears, indicating that both complexes are reduced to water-soluble Cu(I) complexes. Thus, the role of MEA in Cu NWs synthesis is to participate in Cu(II) complexation, preventing the excessive transformation of Cu(I) complexes into Cu_2_O octahedral precipitates. Furthermore, as observed in Figure 5d, MEA co-ordinates with Cu(II) in a tetracoordinate manner in this system. Therefore, we infer that the Cu(I) complex in this system is likely Cu(MEA)_4_(OH), which also adopts a planar configuration.

**Figure 6:**
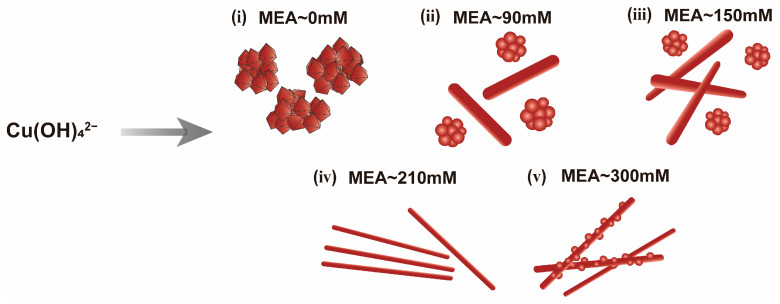
Schematic diagram of the effect of different MEA concentrations on Cu NWs morphology.

Meanwhile, by comparing the SEM images of the products obtained at different MEA concentrations (Figure 2a(A1),2c), it can be observed that, as the MEA concentration increases, the diameter of the Cu NWs initially decreases, then increases after reaching a certain threshold, while their length continuously increases. Based on the results of the MEA concentration experiments, we have illustrated the morphological evolution of Cu NWs as a function of MEA concentration in a schematic diagram (Figure 6). The corresponding diameter and length distributions of the obtained Cu NWs are shown in Figure S2c. Notably, in the MEA concentration experiments, the synthesized Cu NWs almost always exhibited a head with an irregular spherical shape (Figure S5a,b). This observation aligns with the growth mechanism proposed in the literature for Cu NWs synthesized using EDA as a complexing agent, where Cu NWs are grown from spherical seeds [34]. Based on the above trends, we propose that, when the MEA concentration is below 150 mM, MEA preferentially adsorbs onto the sidewalls of the nanowires, leading to the preferential reduction of Cu(MEA)_4_(OH) on the lateral surfaces. This promotes radial growth, resulting in shorter and thicker Cu NWs. When the MEA concentration increases to an optimal range, MEA adsorption becomes more favorable at the ends of the nanowires. As Cu(MEA)_4_(OH) continuously reduces at the wire tips, more MEA accumulates at these sites, facilitating axial growth while suppressing excessive radial expansion. However, when the MEA concentration is excessively high, the abundant MEA enables both axial and radial growth. MEA adsorbed at the wire ends promotes Cu(MEA)_4_(OH) reduction, while surplus MEA still ensures adsorption and reduction along the sidewalls. This leads to simultaneous axial elongation and radial thickening of the Cu NWs, eventually resulting in the formation of nanoparticle-like protrusions along the nanowire surfaces.

**Figure 7:**
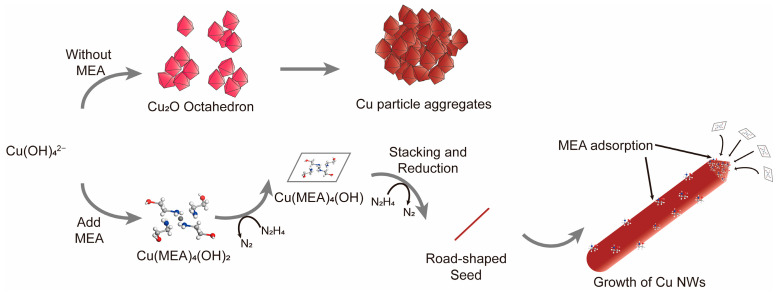
Growth model of Cu NWs.

Based on the above analysis, we propose a growth model for Cu NWs (Figure 7). In the absence of MEA, Cu_2_O octahedra rapidly precipitate and begin to aggregate, ultimately forming agglomerated copper particles upon complete reaction. When an appropriate concentration of MEA is introduced, Cu(II) complexes are rapidly reduced to Cu(I) complexes during the vigorous stirring stage. We hypothesize that, upon the addition of MEA, Cu(II) forms stable complexes with MEA and OH^−^, specifically Cu(MEA)_4_(OH)_2_ and Cu(OH)_4_^2−^. When N_2_H_4_ solution is introduced, intense stirring facilitates the rapid reduction of Cu(MEA)_4_(OH)_2_ and Cu(OH)_4_^2−^ to Cu(MEA)_4_(OH). The reduced solubility of Cu(MEA)_4_(OH) in solution promotes its rapid stacking and subsequent reduction, leading to the formation of ultrafine rod-like seeds. During the subsequent aging stage, MEA gradually adsorbs onto the crystalline seeds, facilitating the elongation and thickening of Cu NWs. Once the Cu NWs in the solution reach saturation, they rapidly precipitate and float to the surface of the solution. Beyond this point, the length of Cu NWs continues to increase until the reaction is complete.

The inhibition of Cu NWs growth after saturation can be attributed to two primary factors. First, as the reaction proceeds, both Cu(II) ions and the reducing agent N_2_H_4_ are gradually consumed, leading to a decrease in the concentration of reactive species available for the reduction process, thereby slowing the growth rate of Cu NWs. Second, the progressive accumulation of Cl^−^ ions in the system results in the formation of stable CuCl_4_^2−^ complexes with residual Cu(II). The formation of this complex significantly reduces the reducibility of Cu(II), further hindering the continued growth of Cu NWs.

It is worth noting that the presence of amine-based complexing agents plays a crucial role in nanowire formation. In this system, MEA co-ordinates with copper ions and facilitates the preferential growth direction of Cu NWs, which is essential for the anisotropic growth into wire-like structures.

Furthermore, to further verify the roles of MEA and N_2_H_4_ in the nanowire synthesis process and to reduce the surface oxidation of Cu NWs, polyethylene glycol (PEG) was introduced during the synthesis of Cu NWs. This decision was based on the observation of the Cu NWs reaction process (Figure 4a–c), where flocculent substances formed after the transparent phase quickly floated to the solution surface. Therefore, PEG200 was selected for addition. The incorporation of PEG200 can create a stable liquid layer, which is expected to capture Cu nanowires and prevent excessive MEA adsorption [39]. Here, the encapsulation effect of PEG200 on Cu NWs is primarily attributed to a physical adsorption mechanism, predominantly governed by steric hindrance. Specifically, PEG molecules, due to their long-chain structure, adsorb onto or entangle with the surface of the nanowires, forming a partial coating layer. This spatial barrier impedes further adsorption of MEA molecules onto the nanowires, thereby suppressing the continued growth of Cu NWs. In this experiment, we selected synthesis conditions that produced rough Cu NWs (Sample C3 in Table 1). After adding N_2_H_4_ for 10 min, PEG200 was slowly introduced along the inner wall, allowing it to float on the surface of the transparent solution (Figure S6b). During the reaction, once Cu NWs were formed, they immediately floated to the interface layer and were encapsulated by PEG200 (Figure S6c). Compared with the initial synthesis system, the resulting Cu NWs exhibited a smooth surface without additional particle growth around them (Figure S6d). At the same time, both the average diameter and length of the nanowires decreased. The resulting Cu NWs had an average diameter of approximately 336.71 nm and an average length of about 28.89 μm (Figure S6e,f). This result indicates that, after the nanowires formed, they floated to the interface and were encapsulated by PEG, preventing further MEA adsorption and thereby inhibiting further growth in both directions.

**Figure 8:**
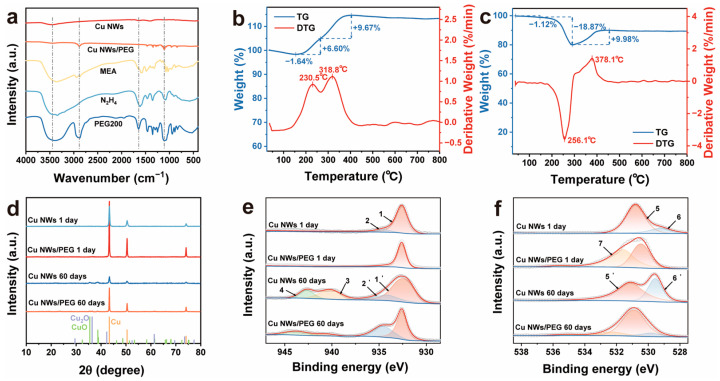
(**a**) FTIR spectra of Cu NWs before and after PEG treatment, along with MEA, N_2_H_4_, and PEG200; (**b**) thermogravimetric analysis (TGA) curve of Cu NWs; (**c**) thermogravimetric analysis (TGA) curve of Cu NWs/PEG; (**d**) XRD patterns of Cu NWs and Cu NWs/PEG after 1 day and 60 days of storage. The reference diffraction patterns at the bottom correspond to Cu (JCPDS 04-0836, orange line), CuO (JCPDS 45-0937, green line), and Cu_2_O (JCPDS 05-0667, purple line); (**e**,**f**) XPS spectra of Cu 2p_3/2_ and O 1s states for Cu NWs and Cu NWs/PEG after 1 day and 60 days of storage.

Fourier transform infrared (FTIR) spectroscopy was performed on the Cu NWs, Cu NWs/PEG, and the precursor materials MEA, N_2_H_4_, and PEG200 (Figure 8a). The Cu NWs exhibited two distinct peaks near 3400 cm^−1^ and 1600 cm^−1^, corresponding to the N-H stretching and bending vibrations of MEA or N_2_H_4_ molecules, respectively. These results align well with our expectations and further validate our hypothesis that MEA facilitates the growth of Cu NWs by adsorption, forming Cu(MEA)_4_(OH), which subsequently reduces to Cu NWs. However, it is noteworthy that, due to the varying time intervals at which the Cu NWs float to the solution surface and become encapsulated by PEG, the resulting Cu NWs/PEG exhibit significant variations in diameter and length. This inconsistency adversely affects the electrical conductivity and optical transparency of the conductive film.

In addition to Fourier transform infrared spectroscopy (FTIR), thermogravimetric (TG) analysis in an air atmosphere was also employed to confirm the presence of PEG on the synthesized Cu NWs. In Figure 8a, we observed that, in addition to a broad peak near 3400 cm^−1^, Cu NWs/PEG also exhibited distinct characteristic peaks at approximately 2800 cm^−1^ and 1110 cm^−1^, corresponding to the C-H stretching vibration and C-O-C stretching vibration, respectively. Comparative TG and DTG analyses of Cu NWs and Cu NWs/PEG show that both samples exhibit an initial weight loss stage, attributed to the removal of water and other solvent molecules (Figure 8b,c) [59]. After this weight loss, Cu NWs undergo two mass increase stages of 6.60% and 9.67%, corresponding to the formation of Cu_2_O and CuO, respectively, followed by a plateau where Cu NWs are completely oxidized. In contrast, Cu NWs/PEG, after the initial weight loss stage, undergoes an 18.87% weight loss and a subsequent 9.98% weight gain, which correspond to PEG200 degradation and Cu NWs oxidation, respectively. These results confirm that PEG200 can successfully encapsulate the surface of Cu NWs.

The Cu NWs and Cu NWs/PEG were simultaneously placed under ambient conditions and exposed to air. X-ray diffraction (XRD) and X-ray photoelectron spectroscopy (XPS) were used to analyze the products after 60 days of exposure in order to investigate their stability. The XPS results were consistent with the XRD findings. Figure 8d presents the obtained XRD patterns, showing that both Cu NWs and Cu NWs/PEG exhibit characteristic peaks of CuO after 60 days of storage, while Cu NWs/PEG also displays characteristic peaks of Cu_2_O. In the XPS analysis, the spectra were referenced to the binding energy of amorphous C 1s at 284.6 eV and fitted using Gaussian curve fitting [60,61]. The Cu 2p_3/2_ and O 1s peaks are shown in Figure 8e and f, respectively. In the spectra, Cu(0) appears as a single peak at 932.6 eV (Peak 1) [61], while Cu(II) exhibits a peak at 934.5 eV (Peak 2), which corresponds to a small amount of Cu(OH)_2_ present on the Cu NWs surface. This Cu(OH)_2_ may originate from reaction intermediates with low crystallinity. After 60 days of storage, the relative content of surface Cu(II) hydroxide (Peak 2′) increases compared to Cu(0) (Peak 1′). Additionally, Cu(II) peaks appear at 940.2 eV and 942.3 eV (Peaks 3 and 4), corresponding to CuO satellite peaks. It is evident that, after 60 days of storage, the intensity of the satellite peaks in the 940–945 eV range is significantly lower for Cu NWs/PEG than for Cu NWs stored under normal conditions, while the relative content of Cu(II) hydroxide is notably higher. This suggests that, after 60 days, the Cu NWs/PEG surface primarily consists of Cu_2_O and copper hydroxycarbonate. Based on the analysis of Figure 8d, the appearance of weak CuO characteristic peaks in the XRD pattern of Cu NWs/PEG after 60 days of storage is likely attributed to the further oxidation of surface-formed Cu_2_O during the measurement. The absence of CuO-related peaks in the XPS spectra is ascribed to the spatially nonuniform oxidation of the sample, as well as slight discrepancies in the timing of the two measurements. The main O 1s peak (Peak 5) is located at 530.8 eV, as shown in Figure 8f. Peaks corresponding to metal oxides are primarily located to the right of the main peak. The CuO peak (Peak 6) is located at 529.2 eV [62], which likely corresponds to a minimal amount of oxidation on the Cu NWs surface during testing. The peak at 531.59 eV (Peak 7) is attributed to the C-O bond in PEG. After 60 days of storage, the CuO peak (Peak 6′) in uncoated Cu NWs shows a significant increase in intensity relative to the O 1s main peak (Peak 5′). These results validate our hypothesis that PEG can effectively encapsulate Cu NWs, significantly reducing their oxidation when exposed to air.

### 3.4. Cu NWs Transparent Conductive Film

**Figure 9:**
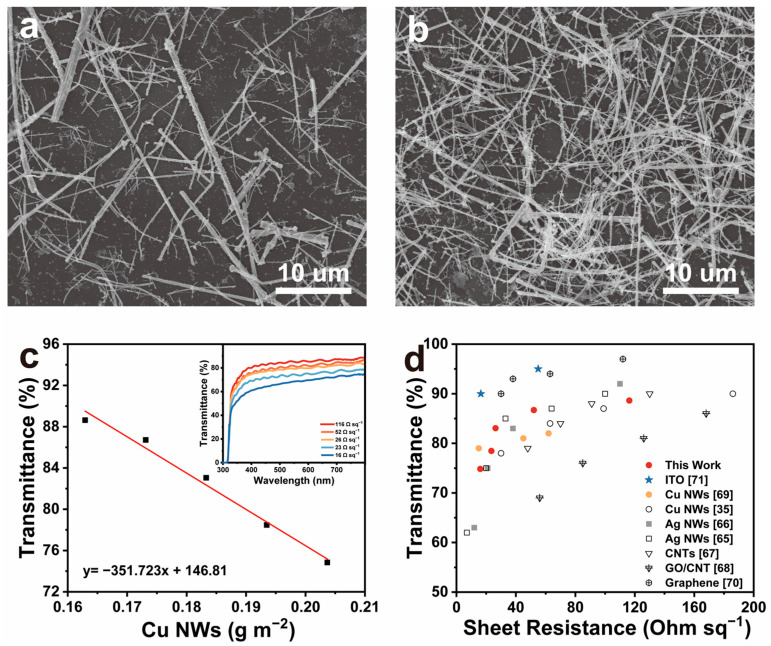
(**a**,**b**) SEM images of Cu NWs transparent conductive films with transmittances of 88.6% and 74.8%, respectively; (**c**) relationship between the transmittance (800 nm) and the Cu NWs density (g m^−2^) in the film (the inset shows the complete transmittance spectra of the films prepared with different Cu NWs densities); (**d**) relationship between transmittance (800 nm) and sheet resistance for Cu NWs, Ag NWs, ITO, graphene, and CNT films.

The vacuum filtration method is a commonly used technique in laboratories for fabricating Cu NWs transparent conductive films due to its simplicity, strong controllability, and low cost [63]. However, during the transfer of Cu NWs from the filter membrane to the substrate under applied pressure, issues such as residual Cu NWs on the filter membrane and incomplete transfer often occur [64]. To address these challenges, we fabricated Cu NWs transparent conductive films using an improved vacuum filtration method (Figure 1). By precisely controlling the concentration and volume of the Cu NWs dispersion, along with the effective area of the filtration membrane, the mass of nanowires deposited per unit area—referred to as the Cu NWs areal density—was accurately determined. Figure 9a,b presents SEM images of Cu NWs films with transmittances of 88.6% and 74.8%, respectively, illustrating the formation of a continuous nanowire network. At lower Cu NWs density (Figure 9a), the nanowires are sparsely distributed on the film, with limited contact and interconnection between them, resulting in a higher optical transmittance (88.6%) and higher sheet resistance (116 Ω sq^−1^). As the Cu NWs density increases (Figure 9b), the nanowires become more densely packed with significantly enhanced inter-nanowire junctions, leading to reduced optical transmittance (74.8%) and decreased sheet resistance (16 Ω sq^−1^). Figure 9c depicts the relationship between the Cu NWs density and transmittance in the films. To achieve a sheet resistance of 26 Ω sq^−1^, a Cu NWs density of only 0.18 g m^−2^ on the PET substrate is required. However, during the dissolution of the MCE filter membrane via acetone and isopropanol immersion, partial loss of Cu NWs occurs, leading to a lower actual Cu NWs content on the final film compared to the theoretical calculation.

The relationship between the transmittance and sheet resistance of the Cu NWs film is shown in Figure 9d. When the sheet resistance of the Cu NWs film is 52 Ω sq^−1^, its transmittance is comparable to that of mechanically coated Ag NWs films [65] and superior to Cu NWs [35], Ag NWs [66], CNTs [67], and GO/CNT [68] films prepared via Meyer rod coating, floating catalyst chemical vapor deposition (CVD), and spray coating. Additionally, compared to Cu NWs films fabricated using the same vacuum filtration method [69], the Cu NWs films obtained in this study after MCE membrane corrosion with acetone and isopropanol exhibit improved performance. However, at higher sheet resistance values, Ag NWs films prepared by coating and CNTs films fabricated by CVD exhibit slightly better performance than Cu NWs films. Furthermore, graphene films [70] demonstrate significantly superior performance compared to Cu NWs films and offer greater flexibility than ITO films [71]. However, graphene films still have some drawbacks compared to Cu NWs films. Under similar optical transmittance conditions, the conductivity of monolayer graphene films is inferior to that of certain Cu NWs films. Moreover, the fabrication of graphene films requires chemical vapor deposition (CVD) at nearly 1000 °C on copper foils, followed by complex transfer and etching processes, which demand sophisticated equipment, meticulous operation, and incur high costs. Therefore, although graphene films offer performance advantages, considering fabrication processes, material costs, and overall feasibility, Cu NWs films represent a more favorable choice.

To evaluate the aging performance of the conductive films, we exposed films prepared with different Cu NWs densities to ambient air at room temperature and measured their sheet resistance on days 1, 10, 20, and 30, as shown in Figure S7. The results reveal that films with higher Cu NWs densities exhibit smaller variations in sheet resistance over time. Notably, the sheet resistance increases sharply on the first day, followed by a gradual stabilization. This initial sharp rise is attributed to the partial surface oxidation of Cu NWs into Cu_2_O or CuO within the first 24 h, leading to the formation of a passivating layer that inhibits further oxidation. As a result, the rate of oxidation slows down significantly after day 1. These findings indicate that the as-prepared Cu NWs films possess a certain degree of oxidation resistance.

We achieved the transfer of Cu NWs from the MCE filter membrane to the PET substrate by dissolving the MCE membrane with acetone and isopropanol. This approach not only removes impurities remaining in the film but also effectively minimizes the loss of Cu NWs that occurs during direct peeling from the filter membrane. Additionally, during the vacuum filtration process, the distribution of Cu NWs on the filter membrane surface may exhibit some degree of nonuniformity. Etching the MCE membrane can partially improve the contact interface between the film and the membrane, allowing the distribution of Cu NWs to be adjusted under the influence of surface tension and other factors. This contributes to enhancing the surface smoothness of Cu NWs-based transparent conductive films [72].

## 4. Conclusions

We replaced copper nitrate with copper chloride and ethylenediamine with monoethanolamine (MEA) and experimentally determined the optimal conditions for synthesizing Cu nanowires (Cu NWs) in the CuCl_2_–MEA–N_2_H_4_ system. The synthesized Cu NWs had an average diameter of approximately 101 nm and a length of about 28 μm. The Cu NWs exhibited a face-centered cubic (FCC) structure and grew along the (110) direction. Raman spectroscopy and TEM analysis of the intermediate reaction solution confirmed the presence of Cu_2_O, indicating that Cu NWs were gradually reduced from Cu(II) to Cu(I) and eventually to Cu(0). MEA concentration experiments and PEG coating experiments further demonstrated that MEA could prevent the excessive transformation of Cu(I) complexes into Cu_2_O octahedral precipitates. Additionally, MEA exhibited an adsorption effect on Cu NWs, influencing their growth by selectively adsorbing onto the ends and sidewalls, thus controlling their morphology based on its concentration. Finally, a Cu NWs transparent conductive film was fabricated using an improved vacuum filtration method, achieving a sheet resistance of 52 Ω sq^−1^ with a transmittance of 86.7%.

## Figures and Tables

**Table 1:** Reaction conditions and corresponding products.

Sample	Temp./°C	NaOH/M	MEA/mM	N_2_H_4_/mM	Product
A1	60	15	210	12.5	Cu NWs
A2	70	15	210	12.5	Mixture of NWs and NRs
A3	80	15	210	12.5	Dominated by NRs
B1	60	7.5	210	12.5	Cu_2_O Tetrahedron
B2	60	10	210	12.5	Mixture of Cu_2_O and NWs
B3	60	12.5	210	12.5	Dominated by NWs
C1	60	15	90	12.5	Mixture of NRs and NPs
C2	60	15	150	12.5	Mixture of NRs and NPs
C3	60	15	300	12.5	Mixture of NWs and NRs
D1	60	15	210	7.5	Dominated by NRs
D2	60	15	210	10	Mixture of NWs and NRs
D3	60	15	210	15	Mixture of NWs and NRs

## Data Availability

Data are contained within the article.

## Supplementary Materials

The following supporting information can be downloaded at: https://www.mdpi.com/article/10.3390/nano15090638/s1. Figure S1: XRD patterns of products synthesized at different NaOH concentrations; Figure S2: (a) Diameter and length distributions of Cu NWs synthesized at different temperatures; (b) Diameter and length distributions of Cu NWs prepared with varying NaOH concentrations; (c) Diameter and length distributions of Cu NWs synthesized with different MEA concentrations (inset: size distribution of copper nanoparticle aggregates formed at corresponding MEA concentrations); (d) Diameter and length distributions of Cu NWs obtained with varying hydrazine hydrate concentrations; Figure S3: XRD pattern of Cu NWs; Figure S4: (a) TEM image of Cu NWs with sheet-like CuO growth; (b) SAED pattern of image a (left parallelogram: CuO, right parallelogram: Cu); Figure S5: (a) SEM detailed image of sample A1; (b) SEM image of the nanowire tips of sample A1; Figure S6: (a) Reaction mixture after stirring; (b) reaction mixture after the addition of PEG200; the black dashed lines indicate the PEG layer; (c) reaction mixture after the completion of the reaction; (d) SEM image of the synthesized Cu NWs; (e) diameter distribution of the synthesized Cu NWs; (f) length distribution of the synthesized Cu NWs; Figure S7: Time-dependent variation of sheet resistance for transparent conductive films with different Cu NWs densities.
