# Supplementary material for: Synthesis of Copper Nanowires Using Monoethanolamine and the Application in Transparent Conductive Films
Source: Nanomaterials (Basel). 2025 Apr 22;15(9):638. doi: 10.3390/nano15090638 (PMC12073782; doi:10.3390/nano15090638)
Supplement: Supplementary file 1 [file nanomaterials-15-00638-s001.zip › nanomaterials-3561064-supplementary.pdf]

nanomaterials

Supporting Information

Synthesis of Copper Nanowires Using Monoethanolamine and The Application in Transparent Conductive Films

Xiangyun Zha [1], Depeng Gong [1], Wanyu Chen [1], Lili Wu [1] and Chaocan Zhang [1,*]

1 School of Materials Science and Engineering, Wuhan University of Technology, Wuhan 430070, China; zxy0108@whut.edu.cn (X.Z.); gdp@whut.edu.cn (D.G.); chenwanyu@whut.edu.cn (W.C.); polym_wl@whut.edu.cn (L.W.)

* Correspondence: polymers@whut.edu.cn (C.Z.)

Supporting Figures

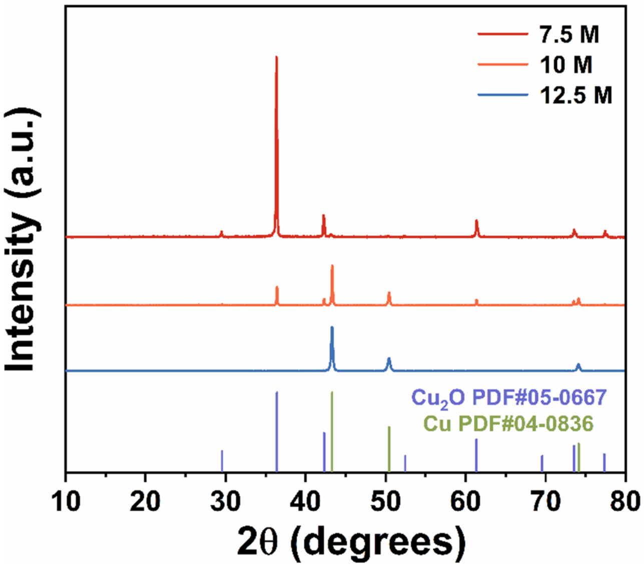

Figure S1. XRD patterns of products synthesized at different NaOH concentrations.

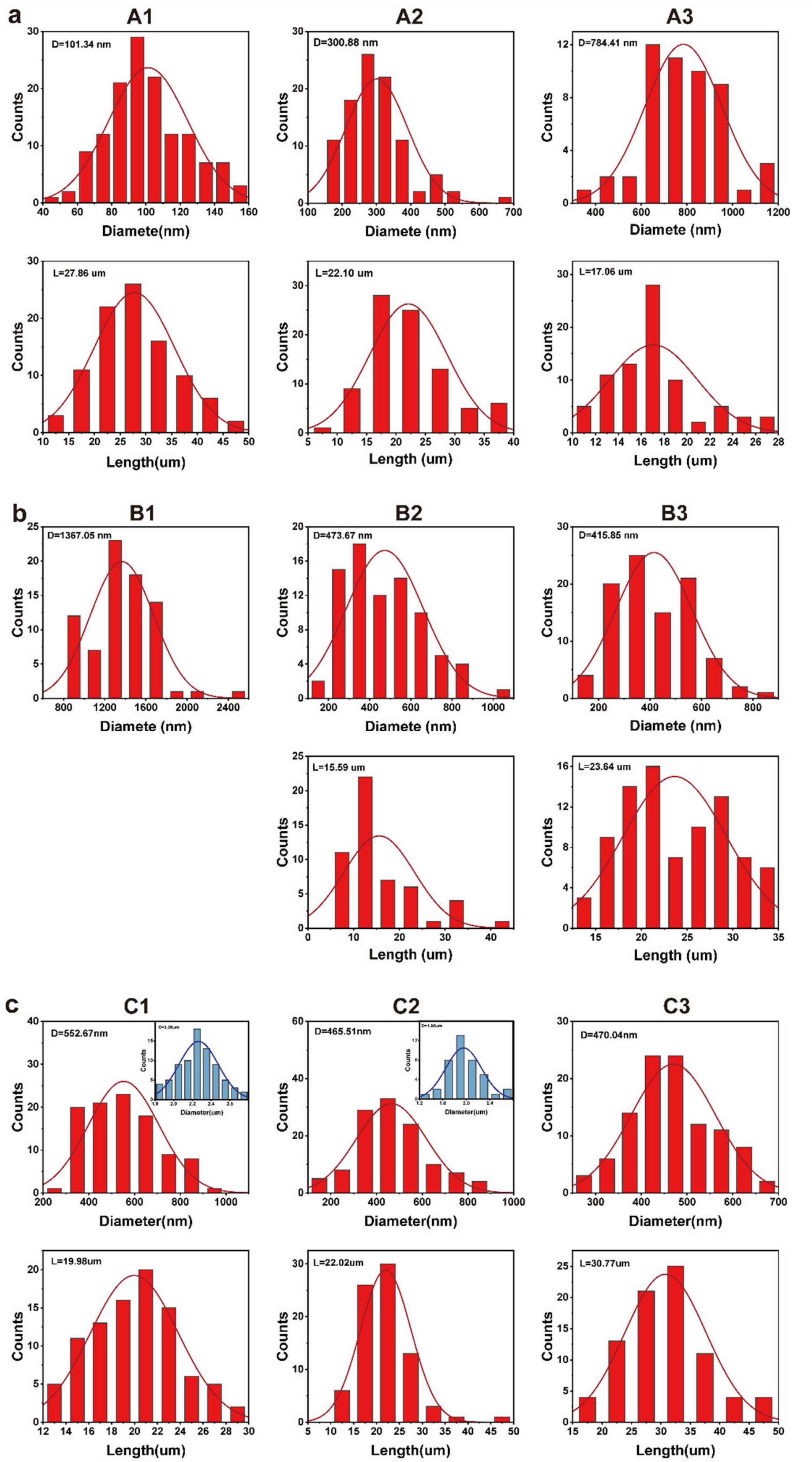
a
A1
D=101.34 nm
Counts
Diamete(nm)
L=27.86 um
Length(um)
A2
D=300.88 nm
Diamete (nm)
L=22.10 um
Length (um)
A3
D=784.41 nm
Diamete (nm)
L=17.06 um
Length (um)
b
B1
D=1367.05 nm
Diamete (nm)
B2
D=473.67 nm
Diamete (nm)
L=15.59 um
Length (um)
B3
D=415.85 nm
Diamete (nm)
L=23.64 um
Length (um)
c
C1
D=552.67nm
Diameter(nm)
L=19.98um
Length(um)
C2
D=465.51nm
Diameter(nm)
L=22.02um
Length(um)
C3
D=470.04nm
Diameter(nm)
L=30.77um
Length(um)

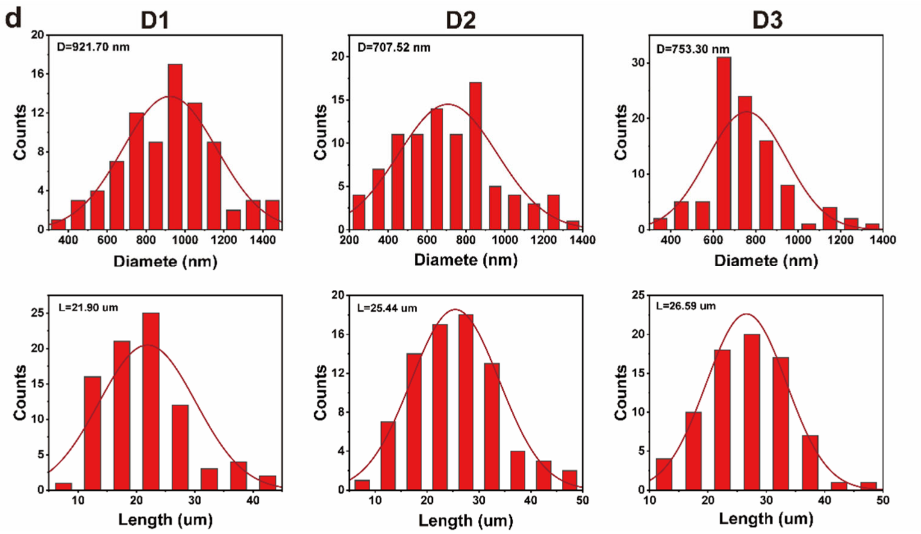

Figure S2. (**a**) Diameter and length distributions of Cu NWs synthesized at different temperatures; (**b**) Diameter and length distributions of Cu NWs prepared with varying NaOH concentrations; (**c**) Diameter and length distributions of Cu NWs synthesized with different MEA concentrations (inset: size distribution of copper nanoparticle aggregates formed at corresponding MEA concentrations); (**d**) Diameter and length distributions of Cu NWs obtained with varying hydrazine hydrate concentrations.

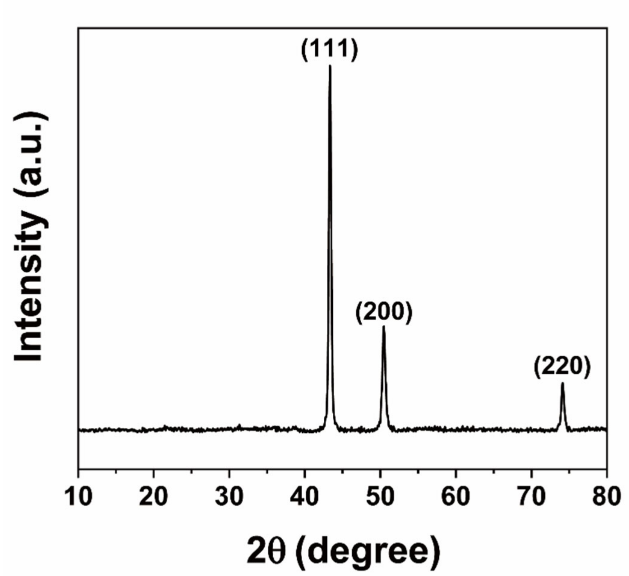

Figure S3. XRD pattern of Cu NWs.

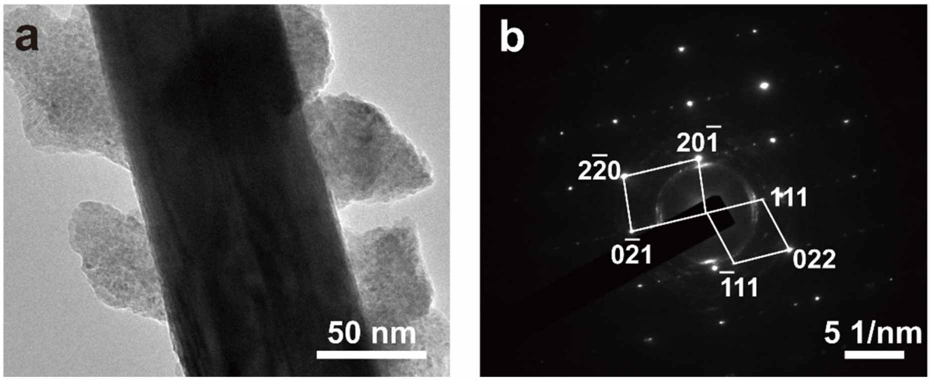

Figure S4. (**a**) TEM image of Cu NWs with sheet-like CuO growth; (**b**) SAED pattern of image a (left parallelogram: CuO, right parallelogram: Cu).

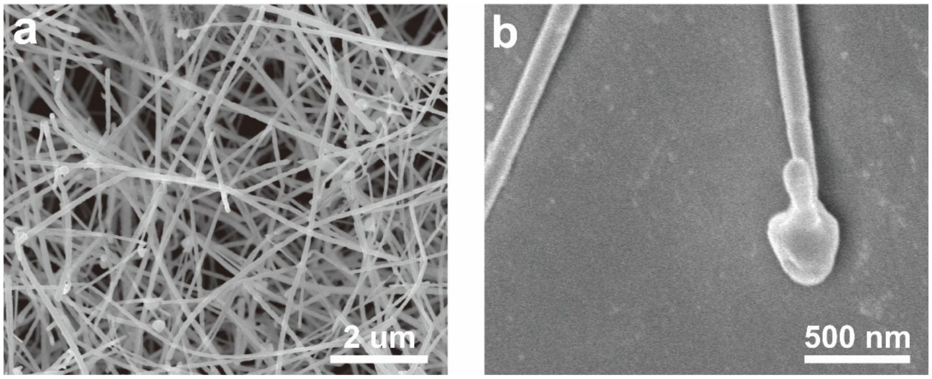

Figure S5. (**a**) SEM detailed image of sample A1; (**b**) SEM image of the nanowire tips of sample A1.

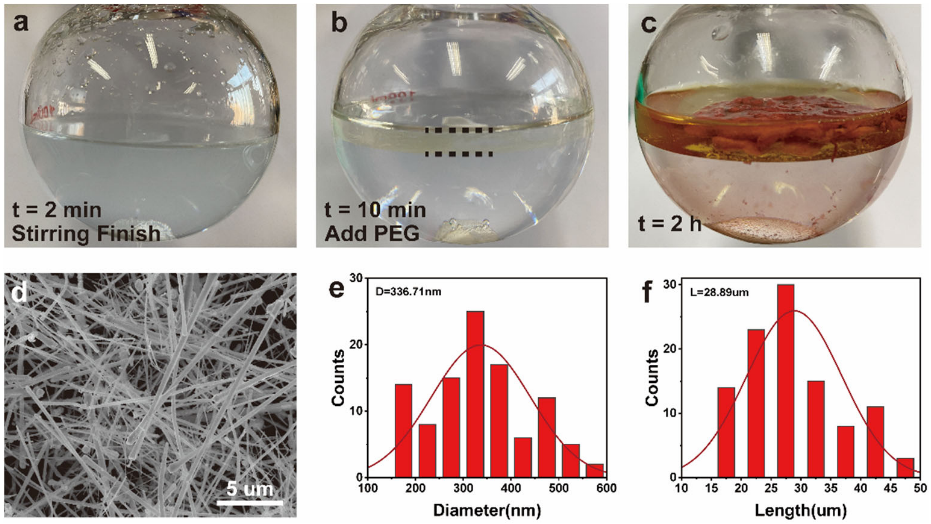

Figure S6. (**a**) Reaction mixture after stirring; (**b**) reaction mixture after the addition of PEG200; the black dashed lines indicate the PEG layer; (**c**) reaction mixture after the completion of the reaction; (**d**) SEM image of the synthesized Cu NWs; (**e**) diameter distribution of the synthesized Cu NWs; (**f**) length distribution of the synthesized Cu NWs.

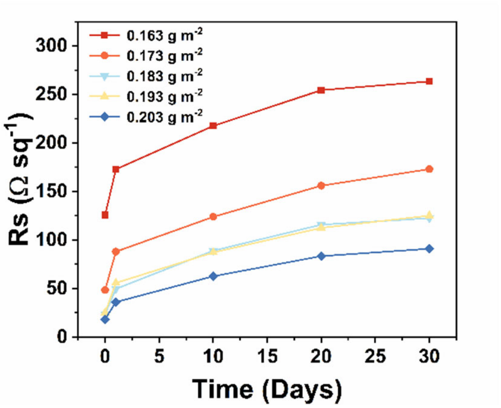

Figure S7. Time-dependent variation of sheet resistance for transparent conductive films with different Cu NWs densities.
